# Supplementary material for: MRIQC: Advancing the automatic prediction of image quality in MRI from unseen sites
Source: PLoS One. 2017 Sep 25;12(9):e0184661. doi: 10.1371/journal.pone.0184661 (PMC5612458; doi:10.1371/journal.pone.0184661)
Supplement: S1 File — All supporting information has been included in one file. (PDF) [file pone.0184661.s001.pdf]

Supporting Information

Block 1 in S1 File Extended caption of Fig 1. An example scan (top) is shown with severe motion artifacts. The reduced contrast between tissues and the ringing intensity waves in the anterior region of the brain in the presented slice suggest a large head movement occurred during acquisition. The green arrows point to signal spillover due to eye movements through the phase-encoding axis (in this case, right-to-left –RL–). Oftentimes, the RL or LR axes are selected for phase-encoding because the signal leakage from the eyeballs does not overlap with brain tissue, as opposed to selecting anterior-posterior directions. However, the red arrows point to signal spillover caused by vessel pulsations. Given the location of the vessel, in this case signal leakage overlaps brain tissue and affects the quality of this image. The phase-encoding axis has less bandwidth and thus, is more sensitive to movement. For that reason, it is generally selected to have the shortest field of view. A second example scan (bottom) shows severe coil artifacts.

Block 2 in S1 File Image acquisition parameters. A table containing all the acquisition parameters is maintained in GitHub: `https://github.com/poldracklab/mriqc/blob/c5019745afb2d8c6ae4230565b668cb34cf21e41/mriqc/data/csv/scan_parameters.tsv`.

Eq 1 in S1 File Definition of the accuracy score (ACC). The accuracy score is calculated as the total of true positive (TP) and true negative (TN) samples over the total of samples:

$$\mathrm{ACC} = \frac{\mathrm{TP} + \mathrm{TN}}{\mathrm{P} + \mathrm{N}}.$$

Eq 2 in S1 File Definition of the sensitivity to the "exclude" class (or recall). The recall is calculated as the total of true positive (TP) samples over the total of positive (manually rated as "exclude") samples:

$$\mathrm{recall} = \frac{\mathrm{TP}}{\mathrm{P}} = \frac{\mathrm{TP}}{\mathrm{TP} + \mathrm{FN}}.$$

Block 3 in S1 File Converting datasets into BIDS The BIDS standard is built on top of well-established file formats such as NIfTI and JSON and uses easy to adopt file and folder naming structure. There are many tools that can help users to convert their data to BIDS. For example, `dcm2niix` (`https://github.com/rordenlab/dcm2niix`) is a robust DICOM and PAR/REC to NIfTI conversion tool that generates BIDS compatible JSON files. It can be used to build custom scripts tailored to a particular IT infrastructure available at one's neuroimaging center. *HeuDiConv* (`https://github.com/nipy/heudiconv`) is a more comprehensive solution that can be configured to take folders of unorganized DICOM files as an input and produce a BIDS compatible file/folder structure as an output. If the data is already in NIfTI file format, and there is a need to preserve disk space, symbolic links can be used to create a view of the dataset compatible with BIDS. For more information about software compatible with BIDS see `http://bids.neuroimaging.io`.

Block 4 in S1 File Running MRIQC The BIDS standard makes MRIQC compatible with almost any input dataset without need for custom settings. Since all the metadata associated to the dataset are found in `bids-data/`, the following example would nicely run without further settings. The second positional argument, `out/` indicates where the outputs will be written, and finally, the `participant` keyword instructs MRIQC to run the first level analysis as specified in BIDS Apps.

Running MRIQC – Participant Level.

```
mriqc bids-data/ out/ participant
mriqc bids-data/ out/ participant --participant_label S001 S002
```

Running MRIQC – Group Level. If the `participant` level was run setting some `--participant_label`, the `group` level is not triggered by default. It can be done manually, pointing the input data folder to the derivatives folder generated with the `participant` level analysis:

```
mriqc out/derivatives/ out/ group
```

Predicting quality. Although the `group` run level will generate a CSV table with the quality label predicted for each sample, it is possible to run the classifier individually:

```
mriqc_clf --load-classifier -X aMRIQC.csv -o mypredictions.csv
```

The default classifier can be replaced by a custom one using:

```
mriqc_clf --load-classifier my_custom_classifier.pklz -X aMRIQC.csv -o mypredictions.csv
```

The documentation website contains more detailed information on how to train custom classifiers, or generate refined results from prediction: http://mriqc.readthedocs.io/en/latest/classifier.html.

Block 5 in S1 File Impact of the labeling protocol and variability sources In an earlier version of this manuscript we reused an existing quality assessment of ABIDE dataset that used a different labeling protocol. We fixed the issue with a second manual assessment of the full ABIDE, now following the labeling protocol. In this second assessment, the expert who rated the ABIDE dataset in first place, re-assessed 601 images. We use those 601 images to evaluate the intra-rater variability using different labeling protocols in Figure A.
In the earlier version of the manuscript using Protocol B for labeling, we introduced an unplanned inter-rater bias since the held-out dataset could not be rated by the same expert who rated the ABIDE dataset. However, by changing the rater from train to test datasets we implicitly controlled the risk of overestimating the classification ACC due to systematic errors (those idiosyncratic to one particular expert) on the labels of the training. This problem was fixed when we used Protocol A and two different experts rated 601 data points of ABIDE (with an overlap of 100 images). An analysis of all these sources of variability is done in Figure B.

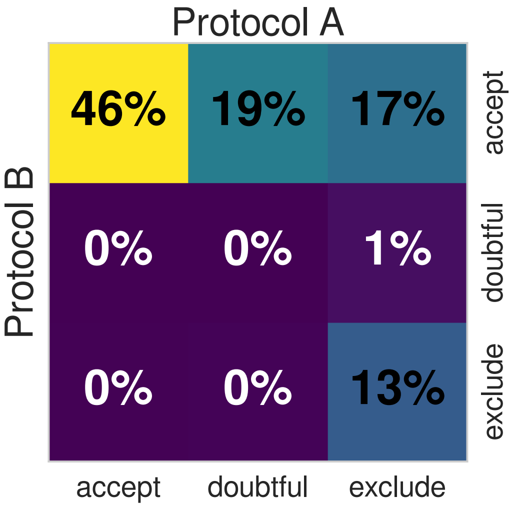

Figure A Intra-rater variability. On the left, the confusion matrix for a single rater using two different labeling protocols is presented. Protocol A corresponds to the procedures used in this work, and described in section Labeling protocol. Protocol B corresponds to the previously existing assessment, that was based on the corrections applied to the surface reconstruction of *FreeSurfer*. The Cohen's Kappa index of agreement for the 3-class labeling was κ=0.29, and that index calculated on binarized labels increases to κ=0.48. Therefore, the labeling protocol defines, in practice, the concept of "quality" for the application at hand. The selection of the labeling procedures requires a trade-off between the time it takes to rate a new data point to increase the training set and the class-noise derived from the unreliability of such rating. The protocol also determines the interpretation of the quality categories by raters. As seen in the figure, only 1% of the images were deemed "doubtful", with a 19% that were accepted with protocol B and rated "doubtful" with protocol A. A similar effect occurs for 17% of the images that were accepted with protocol B (after applying surface corrections) and excluded with protocol A.

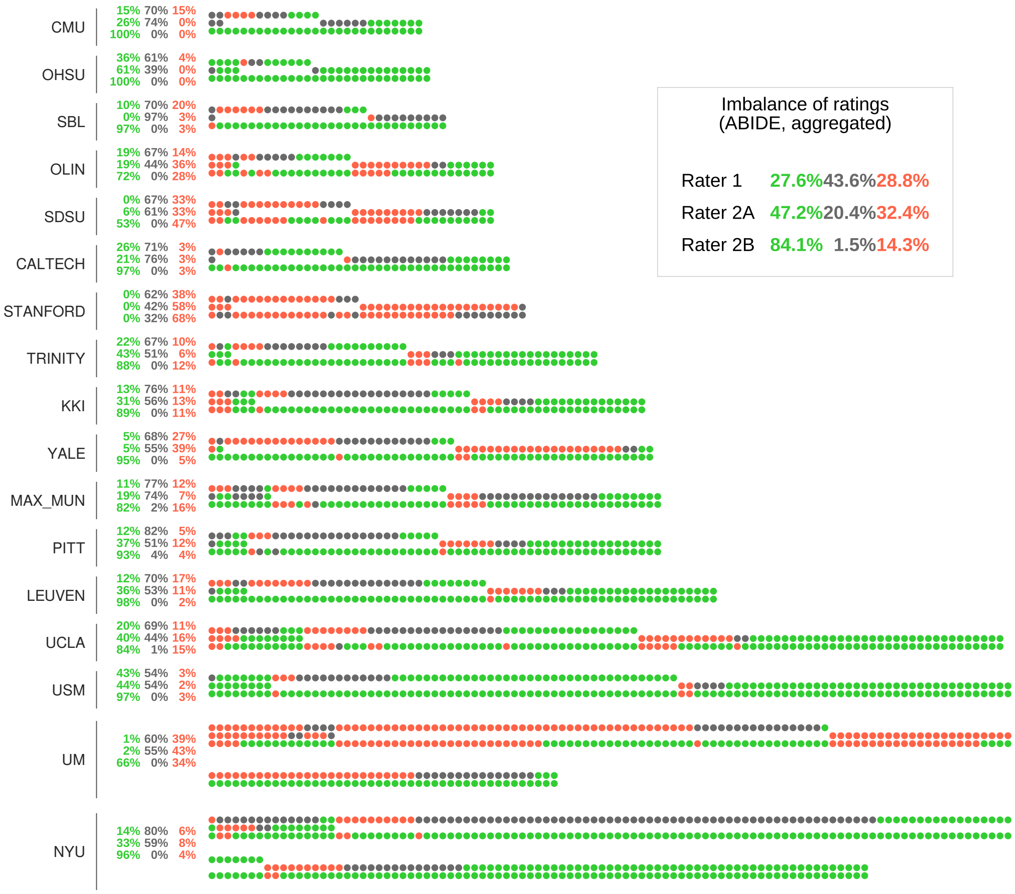

Figure B Quality control of the ABIDE dataset. Info-graphic of the visual assessment of the T1w images of the ABIDE dataset performed by two different experts, split by scanning site. Each scanning site has one stripe with three rows of colored circles, except sites with large samples where the ratings are wrapped in two stripes. Each circle represents the one rating of one image by one of the experts, with the color encoding the quality label (green is "accept", gray is "doubtful", red is "exclude" and missing dots represent missing ratings). Second and third rows are ratings from the same expert, using the two different protocols A and B. Protocol A corresponds to that used in this work to train and test the classifier. Protocol B is an alternative labeling protocol that used the surfaces reconstructed by *FreeSurfer* after manually correcting errors. A perfect agreement between ratings occurs when the three circles of a column show the same color (for example, the first participant of the "OLIN" scanning site). Some images yielded no agreement across raters and protocols (e.g. the first participant in the "MAX_MUN" sample). For the intra-rater reliability (2nd and 3rd row), a consistent direction of discrepancy is observed, whereby images with poorer quality were considered good after manual corrections in the corresponding protocol (3rd row). Next to each site label, the frequency of quality labels is reported, using the same color code. The aggregated (all sites of ABIDE) frequencies are presented in the top-right box.

Block 6 in S1 File The idiosyncratic ghost of DS030. We found the ghosting artifact of *DS030* (see Fig 7B) in around 18% of the images. Since there are no examples in the training set with a comparable ghosting artifact, we question what the performance on the held-out dataset would have been, had we removed those artifacts from the test sample. In an exploratory experiment, we removed those data points (file `mriqc/data/csv/manual_qc/ds030_ghosts_x.csv` in the GitHub repository) from the test set, and run the evaluation experiment again. The results are summarized in Table 1 in S1 File.

Table 1 in S1 File Evaluation on the held-out dataset, after removal of images showing the ghosting artifact. The model cross-validated on the ABIDE dataset performs with AUC=0.832 and ACC=87% on *DS030*.

A. Confusion matrix

		Predicted		
		accept	reject	total
True	accept	175	14	189
	reject	15	13	28
	total	190	27	

B. Performance report

	precision	recall	F1 score	support
accept	0.92	0.93	0.92	189
exclude	0.48	0.46	0.47	28
avg / total	0.86	0.87	0.87	217

Block 7 in S1 File Reproducing the experiments All the experiments presented are run using *Singularity* [29] on two different high performance computing clusters.

Extracting IQMs The extraction of IQMs was run on the *Sherlock* cluster at Stanford University, CA, USA, using the following command lines:

```
# For ABIDE
singularity exec -B $DATA_HOME:/mnt \
    $SINGULARITY_IMAGES/poldracklab_mriqc_0.9.6-2017-06-03-99db97c9be2e.img \
    mriqc /mnt/ABIDE output/ participant \
    --verbose-reports --n_procs 16 --ants-nthreads 8 -m T1w -f --mem_gb 64000

# For DS030
singularity exec -B $DATA_HOME:/mnt \
    $SINGULARITY_IMAGES/poldracklab_mriqc_0.9.6-2017-06-03-99db97c9be2e.img \
    mriqc /mnt/DS030 output/ participant \
    --verbose-reports --n_procs 16 --ants-nthreads 8 -m T1w -f --mem_gb 64000
```

Split scheme for cross validation The experiment presented in What data split should be used in cross-validation? was run on the *Sherlock* cluster at Stanford University, CA, USA, using the following command lines:

```
# [Cross-validation] Inner loop: K-fold; Outer: LoSo
singularity exec \
    $SINGULARITY_IMAGES/poldracklab_mriqc_0.9.7.clf-2.9-2017-06-21-f32ea06e4a73.img \
    mriqc_clf --train --test --log-file --nested_cv --cv kfold -v --njobs 16

# [Cross-validation] Inner loop: LoSo; Outer: LoSo
singularity exec \
    $SINGULARITY_IMAGES/poldracklab_mriqc_0.9.7.clf-2.9-2017-06-21-f32ea06e4a73.img \
    mriqc_clf --train --test --log-file --nested_cv -v --njobs 16

# [Cross-validation] Inner loop: LoSo; Outer: K-fold
singularity exec \
    $SINGULARITY_IMAGES/poldracklab_mriqc_0.9.7.clf-3.2-2017-06-25-52954107a754.img \
    mriqc_clf --train --test --log-file --nested_cv_kfold --cv kfold -v --njobs 16

# [Cross-validation] Inner loop: LoSo; Outer: K-fold
singularity exec \
    $SINGULARITY_IMAGES/poldracklab_mriqc_0.9.7.clf-3.2-2017-06-25-52954107a754.img \
    mriqc_clf --train --test --log-file --nested_cv_kfold -v --njobs 16
```

Nested cross-validation evaluation The experiment presented in Model evaluation and selection was run on the *Wrangler* supercomputer at the Texas Advanced Computer Center, University of Texas, Austin, TX, USA

```
# SVC linear
singularity exec \
    $SINGULARITY_IMAGES/poldracklab_mriqc_0.9.7.clf-3.2-2017-06-25-52954107a754.img \
    mriqc_clf --train --test --log-file -v --nested_cv -M svc_lin --njobs 24

# SVC radial basis function
singularity exec \
    $SINGULARITY_IMAGES/poldracklab_mriqc_0.9.7.clf-3.2-2017-06-25-52954107a754.img \
    mriqc_clf --train --test --log-file -v --nested_cv -M svc_rbf --njobs 24

# RFC
singularity exec \
    $SINGULARITY_IMAGES/poldracklab_mriqc_0.9.7.clf-3.2-2017-06-25-52954107a754.img \
    mriqc_clf --train --test --log-file -v --nested_cv -M rfc --njobs 24
```

Evaluation on the held-out dataset The experiment presented in Evaluation on held-out data was run on the *Wrangler* supercomputer at the Texas Advanced Computer Center, University of Texas, Austin, TX, USA

```
# Model selection and evaluation on DS030
singularity exec \
    $SINGULARITY_IMAGES/poldracklab_mriqc_0.9.7.clf-3.3-2017-07-03-471ff1e21539.img \
    mriqc_clf --train --test --log-file -v --perm 1000 -M rfc --njobs 24

# Evaluation on DS030 without the ghosting artifact
MRIQC_PATH=/usr/local/miniconda/lib/python3.6/site-packages/mriqc; \
singularity exec \
    $SINGULARITY_IMAGES/poldracklab_mriqc_0.9.7.clf-3.3-2017-07-03-471ff1e21539.img \
    mriqc_clf \
    --load-classifier $MRIQC_PATH/data/mclf_run-20170703-190702_mod-rfc_ver-0.9.7.clf-3.3_class-2_cv-
        loso_data-train_estimator.pklz \
    --test $MRIQC_PATH/data/csv/x_ds030-0.9.6-2017-06-03-99db97c9be2e.csv \
           $MRIQC_PATH/data/csv/y_ds030_noghost.csv \
    --log-file -v --njobs 24
```

Report the recall within the nested cross-validation experiment The experiment presented in Evaluation on held-out data was run on the *Wrangler* supercomputer at the Texas Advanced Computer Center, University of Texas, Austin, TX, USA

```
singularity exec \
    $SINGULARITY_IMAGES/poldracklab_mriqc_0.9.7.clf-3.5-2017-08-09-f55fc2488cf3.img \
    mriqc_clf --train --test --log-file -v --nested_cv -M rfc --njobs 24
```
